# Effectiveness of agricultural waste in the enhancement of biological denitrification of aquaculture wastewater

**DOI:** 10.7717/peerj.13339

**Published:** 2022-04-28

**Authors:** Shuwei Gao, Wangbao Gong, Kai Zhang, Zhifei Li, Guangjun Wang, Ermeng Yu, Yun Xia, Jingjing Tian, Hongyan Li, Jun Xie

**Affiliations:** 1 Key Laboratory of Tropical and Subtropical Fishery Resource Application and Cultivation, Pearl River Fisheries Research Institute, Chinese Academy of Fishery Sciences, Guangzhou, Guangdong, China; 2 Guangdong Ecological Remediation of Aquaculture Pollution Research Center, Guangzhou, Guangdong, China; 3 National Demonstration Center for Experimental Fisheries Science Education, Shanghai Ocean University, Shanghai, China

**Keywords:** Solid carbon sources, Kinetic, Denitrification, Agricultural wastes, Aquaculture wastewater

## Abstract

Nitrogen pollution in aquaculture wastewater can pose a significant health and environmental risk if not removed before wastewater is discharged. Biological denitrification uses external carbon sources to remove nitrogen from wastewater; however, these carbon sources are often expensive and require significant energy. In this study, we investigated how six types of agricultural waste can be used as solid carbon sources in biological denitrification. Banana stalk (BS), loofah sponge (LS), sorghum stalk (SS), sweet potato stalk (SPS), watermelon skins (WS) and wheat husk (WH) were studied to determine their capacity to release carbon and improve denitrification efficiency. The results of batch experiments showed that all six agricultural wastes had excellent carbon release capacities, with cumulative chemical oxygen demands of 37.74–535.68 mg/g. During the 168-h reaction, the carbon release process followed the second-order kinetic equation and Ritger-Peppas equation, while carbon release occurred *via* diffusion. The kinetic equation fitting, scanning electron microscopy, and Fourier transform infrared spectroscopy results showed that LS had the lowest *c_m_* and the maximum *t_1/2_* values and only suffered a moderate degree of hydrolysis. It also had the lowest pollutant release rate and cumulative chemical oxygen demand, as well as the most efficient removal of total phosphorous (TP) and total nitrogen (TN). Therefore, we concluded that LS has the lowest potential risk of excess carbon release and capacity for long-lasting and stable carbon release. The WS leachate had the highest TN contents, while the SPS leachate had the highest TP content. In the 181-h denitrification reaction, all six agricultural wastes completely removed nitrate and nitrite; however, SS had the highest denitrification rate, followed by LS, WH, BS, SPS, and WS (2.16, 1.35, 1.35, 1.34, 1.34, and 1.01 mg/(L**·**h), respectively). The denitrification process followed a zero-order and first-order kinetic equation. These results provide theoretical guidance for effectively selecting agricultural waste as a solid carbon source and improving the denitrification efficiency of aquaculture wastewater treatment.

## Introduction

Aquaculture is the primary source of aquatic products due to the decrease in wild fishery resources. In 2018, the total output of aquatic products in China expanded to 47.6 million tons, and accounted for 58% of global aquaculture production (Food and Agriculture Organization of the United Nations (FAO), 2020). Intensive culture methods generally use significant quantities of feed. However, approximately 75% of the nitrogen in the feed is retained in aquaculture water, mainly as soluble nitrogen, owing to low feed-utilization rates during cultivation (Fang et al., 2017). At the same time, fish generate a substantial amount of excreta, which accumulates in water and negatively affects the quality of aquatic products, as well as causes serious environmental problems. The Second National Pollution Sources survey showed that there was a total of 99,100 tons of nitrogen emissions from aquaculture in 2017 (Ministry of Ecology & Environment of the People’s Republic of China, 2020). Therefore, to protect the environment and human health, it is important to remove nitrogen from aquaculture wastewater before its discharge into surrounding waters.

Biological denitrification is considered to be the most effective and promising technology for removing nitrogen from wastewater (John, Krishnapriya & Sankar, 2020). It employs denitrifying bacteria, which use NO_3_^−^-N as terminal electron acceptors and organic substances as electron donors and energy to maintain the growth of microorganisms and remove nitrogen from water in the form of N_2_ (Liu et al., 2017b). The denitrification rate is highly dependent on the carbon–nitrogen ratio (C/N) of the substrates used (Liu et al., 2017a). However, the C/N ratio of aquaculture wastewater is usually below four and does not contain enough carbon to ensure sufficient electron donors for the denitrification process. This limits the effect of denitrification (Ryu & Lee, 2009; Khursheed et al., 2018).

Conventionally, external carbon sources, such as methanol, acetic acid, and glucose, are added to the wastewater to enhance the denitrification process (Huang et al., 2012; Zheng et al., 2018). However, these carbon sources are generally expensive and have high energy and operating requirements. In contrast, using agricultural waste as a carbon source has significant economic advantages and is highly efficient (Huang, 2017). To date, several agricultural waste types, such as corncob (Liu et al., 2017a), peanut shell (Xiong et al., 2019), rice straw (Zhou et al., 2019), rice husk (Fu et al., 2014), and wheat straw (Tao et al., 2020), have been investigated for their application as solid carbon sources to denitrify municipal sewage and industrial wastewater. These agricultural waste types have excellent carbon release abilities with chemical oxygen demands (CODs) of 100–250 mg/g and promising denitrification potentials of 0.42–0.67 mg NO_3_^−^-N/h (Xiong et al., 2020; Ling et al., 2021).

Extensive research has confirmed that adding agricultural waste to municipal sewage and industrial wastewater can effectively improve denitrification efficiency. However, it remains unclear whether the impact of agricultural waste on denitrification in aquaculture wastewater treatment is fast and long-lasting. Furthermore, the six kinds of agricultural wastes selected in this study have a large output, and there are few reports in previous studies. A study by Li, Sun & Song (2019) has shown that maize cobs enhance nitrogen removal from effluents of marine recirculating aquaculture system in saline constructed wetlands. Therefore, we hypothesize that agricultural waste can promote denitrification efficiency during aquaculture wastewater treatment. The main objective of this study was to investigate the feasibility of utilizing six common agricultural wastes as external carbon sources to enhance denitrification efficiency in aquaculture wastewater treatment. The investigated wastes were banana stalk (BS), loofah sponge (LS), sorghum stalk (SS), sweet potato stalk (SPS), watermelon skins (WS) and wheat husk (WH). Each agriculture waste was subjected to a comprehensive investigation of the organics released, surface properties, carbon release kinetics, and denitrification kinetics using batch experiments. These results provide guidance for the selection of agricultural waste as an additional carbon source to improve denitrification efficiency in the treatment of aquaculture wastewater.

## Materials and Methods

### Materials

Four common agricultural wastes, LS, SS, SPS, and WH, were obtained from a household in the rural areas in Binzhou, China, while BS and WS were obtained from a rural area in Guangzhou, China. The BS, SS, SPS, LS and WS were trimmed into approximately 1 cm^3^ cubes in the laboratory, while the WH was not treated. After this, they were washed twice with deionized water to remove surface dust and other impurities and dried at 60 °C in an oven until the weight was constant (Xiong et al., 2020). Then, they were stored into sealed bags for use in subsequent experiments.

### Carbon source release experiment

Five grams of each agricultural waste was added to an Erlenmeyer flasks (1,000 mL), along with 900 mL of ultrapure water (Yang et al., 2015). The Erlenmeyer flasks were sealed with rubber stoppers and left to stand at room temperature (22 ± 2 °C) without shaking and under a light intensity of 355 ± 31 Lux. This process was repeated to produce three replicates of each waste type. Five milliliter water samples from the Erlenmeyer flasks were taken after 1, 2, 4, 8, 12, 24, 48, 72, 96, 120, 144 and 168 h to measure the concentrations of COD, total nitrogen (TN), and total phosphorus (TP). After each sampling, the same volume (5 mL) of ultrapure water was added to the Erlenmeyer flasks. Second-order and Ritger-Peppas (R-P) kinetic equations were fitted to the carbon release process of each agricultural waste.

The second-order kinetic equation is as follows:

(1) $${\rm d}c/{\rm d}t = kc^2$$

where *c* is the cumulative COD concentration at time *t*, mg/(g**·**L); *k* is the constant of carbon release rate; and *t* is time, h. Equation (1) is equivalent to the following equation:

(2) $${\rm 1}/c-{\rm 1}/c_{\rm m} = k/t$$

where *c*_*m*_ is the ultimate cumulative COD concentration, mg/(g**·**L). When *K* = 1/*k*, then Eq. (2) can be written as follows:

(3) $${\rm 1}/c-{\rm 1}/c_{\rm m} = K\;t$$

where *K* is the mass transfer coefficient, mg/(h**·**g**·**L), which reflects the resistance encountered during the release process. Equation (3) is equivalent to the following equation:

(4) $$K = c_{\rm m} / t_{1/2}$$

where *t*_1/2_ is the time required for the concentration of carbon to decrease to half of its maximum concentration, h.

The R-P kinetic equation is written as follows:

(5) $$M_t / M_\infty = k t^{\rm n}$$

where *M*_*t*_ is the cumulative COD concentration at time *t*, mg/(g**·**L); *M*_*∞*_ is the ultimate cumulative COD, mg/(g**·**L); *k* is the constant of carbon release; and *n* is the carbon release index, which represents the mechanism of carbon release. When *n* is less than 0.45 or greater than 0.89, the main mechanisms of carbon release are diffusion and disintegration, respectively. When *n* is between 0.45 and 0.89, the main mechanisms of carbon release are diffusion and disintegration both (Jing et al., 2019).

### Biological denitrification experiment

Synthetic aquaculture wastewater was prepared according to the method of Li et al. (2019) and Zhu et al. (2015), and its composition is shown in Table 1. The mass concentration of NO_3_^−^-N in the wastewater was 50 mg/L. Inoculation denitrification sludge was collected from a pond with a culture history at the Pearl River Fisheries Research Institute in Guangzhou, China. The collected sludge black and viscous. It was filtered with gauze (16 mesh) to remove impurities, washed twice with ultrapure water, and then concentrated (100 r/min, 2 min) (Xu, Dai & Chai, 2019).

**Table 1:** Composition of synthetic aquaculture wastewater.

Component	Concentration (mg/L)	Manufacturers	Purity specification
KNO_3_	360.0	Shanghai Macklin Biochemical Co., Ltd	Analytical Reagent
NH_4_Cl	21.2	Shanghai Macklin Biochemical Co., Ltd	Analytical Reagent
NaNO_2_	12.3	Shanghai Macklin Biochemical Co., Ltd	Analytical Reagent
KH_2_PO_4_	44.0	Shanghai Macklin Biochemical Co., Ltd	Analytical Reagent
K_2_HPO_4_	78.0	Shanghai Macklin Biochemical Co., Ltd	Analytical Reagent
MgSO_4_·7H_2_O	44.0	Shanghai Macklin Biochemical Co., Ltd	Analytical Reagent
KCl	37.0	Shanghai Macklin Biochemical Co., Ltd	Analytical Reagent
Trace element 0.2% (V/V)			
EDTA	640.0	Shanghai Macklin Biochemical Co., Ltd	Analytical Reagent
FeSO_4_·7H_2_O	550.0	Shanghai Macklin Biochemical Co., Ltd	Analytical Reagent
ZnSO_4_·7H_2_O	230.0	Shanghai Macklin Biochemical Co., Ltd	Analytical Reagent
MnSO_4_·H_2_O	340.0	Shanghai Macklin Biochemical Co., Ltd	Analytical Reagent
CuSO_4_·5H_2_O	75.0	Shanghai Macklin Biochemical Co., Ltd	Analytical Reagent
Co (NO_3_)_2_·6H_2_O	47.0	Shanghai Macklin Biochemical Co., Ltd	Analytical Reagent
(NH_4_)_6_Mo_7_O_24_·4H_2_O	25.0	Shanghai Macklin Biochemical Co., Ltd	Analytical Reagent

**Note:**

“Trace element 0.2% (V/V)” indicates that the volume ratio of trace elements to all elements is 0.2%.

Five grams of selected agricultural waste, 50 mL of concentrated denitrification sludge, and 900 mL of synthetic aquaculture wastewater were placed in Erlenmeyer flasks (1,000 mL) (Xiong et al., 2019). An Erlenmeyer flask without agricultural waste addition was used as a control group, and three flasks were prepared for each agricultural waste. Five milliliters of water samples were collected after 0, 4, 8, 13, 18, 23, 30, 37, 49, 61, 85, 133 and 181 h to measure the concentrations of COD, TN, NO_3_^−^-N, NO_2_^−^-N and TP. After each sampling was taken, the same volume of synthetic aquaculture wastewater were added to the Erlenmeyer flasks. The experiments were carried out at room temperature (22 ± 1 °C) and at a light intensity of 355 ± 31 Lux.

### Kinetic equation of denitrification

The NO_3_^−^-N variation curves (the stage of NO_3_^−^-N concentration above 0.5 mg/L) for the six agricultural wastes followed the zero-order and first-order kinetic equations.

The zero-order kinetic equation is as follows:

(6) $$c_t/c_0 = k_1t$$

where *c*_*t*_ is the NO_3_^−^-N concentration of the influent at time *t*, mg/L; *c*_*0*_ is the initial NO_3_^−^-N concentration, mg/L; *t* is time, h; and *k*_*1*_ is the zero-order rate constant (Wen et al., 2010). The first-order kinetic equation is as follows:

(7) $${\rm ln} (c_0/c_t) = k_2t$$

where *k*_*2*_ is the first-order rate constant (Contrera et al., 2014).

### Characterization method of carbon source structure

The surface morphology of the six types of agricultural waste (dried at 60 °C) were determined before and after leaching using scanning electron microscopy (SEM) (QUANTA 250, Servicebio Co., China). The absorption intensity of the agricultural waste at different wavelengths of infrared light represented different the functional groups. To analyze the functional groups of each agricultural waste before and after soaking, the samples were measured using Fourier transform infrared (FTIR) spectroscopy (Model FTIR-650, Servicebio Co., Hubei, China) at a rate of 16 times/min, in the range of 0–4,000 cm^−1^.

### Analysis methods

All water samples were filtered using a 0.45 µm filter (PALL, China) before the determination of NO_3_^−^-N, and NO_2_^−^-N. The concentrations of COD, TN, TP, NO_3_^−^-N, and NO_2_^−^-N were determined according to the methods of Zhang et al. (2020) and Tu et al. (2010). The COD concentration was determined using the acidic potassium dichromate oxidation method (HACH heating system, DR 900, DRB 200; HACH Co., Loveland, CO, USA); TN and TP concentrations were measured using the potassium persulfate digestion method; NO_3_^−^-N and NO_2_^−^-N concentrations were measured using the phenol disulfonic acid and Griess-Saltzman methods, respectively.

All data were analyzed by one-way analysis of variance (ANOVA) and presented as mean values ± SD (standard deviation). Significant differences in the means between treatments were determined by Duncan’s multiple range tests. Probabilities of *P* < 0.05 were considered significant. GraphPad Prism 8.0.2 and Origin 2019b were used to plot the data.

## Results

### Carbon release performance

#### Amount of released carbon

**Figure 1:**
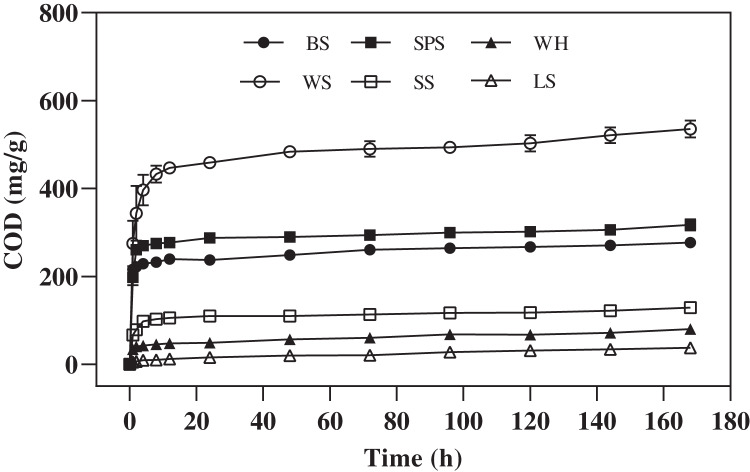
COD released of six agricultural wastes. Each data point indicates the average COD release of each agricultural waste with time. BS, banana stalk; SS, sorghum stalk; SPS, sweet potato stalk; WH, wheat husk; LS, loofah sponge; WS, watermelon skins.

In this experiment, the amount and change trend of carbon release of each agricultural waste mixed with ultrapure water are shown in Fig. 1, the different agricultural wastes had similar trends in COD release, but the amounts of COD released were significantly different (*P* = 0.0000–0.0004). The cumulative concentration of COD released by WS, SPS, BS, SS, WH, and LS were 535.68 ± 15.59, 317.04 ± 10.75, 276.72 ± 1.18, 129.12 ± 1.86, 79.68 ± 6.07, and 37.74 ± 3.61 mg/g, respectively. These values show that the COD release of WS and SPS was significantly higher than that of the other four agricultural wastes (*P* = 0.0000–0.0004). The COD release process of WS, SPS, BS, and SS included two main stages. In the first 4 h, the COD concentrations of WS, SPS, BS, WH, and SS increased rapidly, and between 4 and 168 h, the COD release rates gradually decreased and finally stabilized at very low levels. However, the COD release of LS increased slowly and then, maintained a stable release rate throughout the carbon release process.

#### Kinetic equation of carbon release

The released CODs were fitted using kinetic Eqs. (2) and (5). The kinetic equation along with the correlation coefficients (*R*^*2*^), carbon release indexes (*n*), mass transfer coefficient (*K*), and other relevant parameters are listed in Table 2. The carbon release processes of the six agricultural wastes were in accordance with the second-order reaction and R-P kinetic equation. According to the fitting results of the R-P calculation, the carbon release indices were all less than 0.45, meaning that the selected agricultural wastes released small molecular organic matter that was transferred to the solution through diffusion. It also indicates that the lignocellulosic framework was stable. The *t*_*1/2*_ values of the agricultural wastes ranged from 0.22 to 3.65, indicating that they could release large amounts of organic matter in a short period of time. BS, WS, and SPS had the highest *c*_*m*_ values (312.50, 625.00, and 370.37, respectively), followed by SS and WH (142.86 and 72.99, respectively). LS had the lowest *c*_*m*_ value (25.28), resulting in the lowest risk of excessive effluent carbon release in the initial denitrification process. In addition, for LS, the relative maximum *t*_*1/2*_ (3.65) indicated that the carbon release process was more uniform and conducive to lasting carbon release.

**Table 2:** Kinetic fitting equation of carbon release process.

Carbon source	Second-order fitting equation						Ritger-Peppas fitting equation		
	Equation	R^2^	*c*_*m*_	*K*	*t*_1/2_		Equation	R^2^	*n*
BS	1/*c* = 0.0007/*t* + 0.0032	0.61	312.50	1,428.57	0.22		*M*_*t*_/*M*_*∞*_ = 0.7687 *t*^0.05^	0.98	0.05
SPS	1/*c* = 0.0013/*t* + 0.0027	0.93	370.37	769.23	0.48		*M*_*t*_/*M*_*∞*_ = 0.8028 *t*^0.04^	0.95	0.04
WH	1/*c* = 0.0118/*t* + 0.0137	0.72	72.99	84.75	0.86		*M*_*t*_/*M*_*∞*_ = 0.4266 *t*^0.14^	0.93	0.14
WS	1/*c* = 0.0014/*t* + 0.0016	0.97	625.00	714.29	0.87		*M*_*t*_/*M*_*∞*_ = 0.7225 *t*^0.06^	0.91	0.06
SS	1/*c* = 0.0056/*t* + 0.007	0.96	142.86	178.57	0.80		*M*_*t*_/*M*_*∞*_ = 0.6248 *t*^0.09^	0.87	0.09
LS	1/*c* = 0.1426/*t* + 0.0391	0.88	25.58	7.01	3.65		*M*_*t*_/*M*_*∞*_ = 0.1174 *t*^0.40^	0.99	0.40

**Note:**

Each data used in the fitting equation is the average value of COD released by each agricultural waste within 168h. BS, banana stalk; SS, sorghum stalk; SPS, sweet potato stalk; WH, wheat husk; LS, loofah sponge; WS, watermelon skins.

### Release characteristics of secondary pollutants

**Figure 2:**
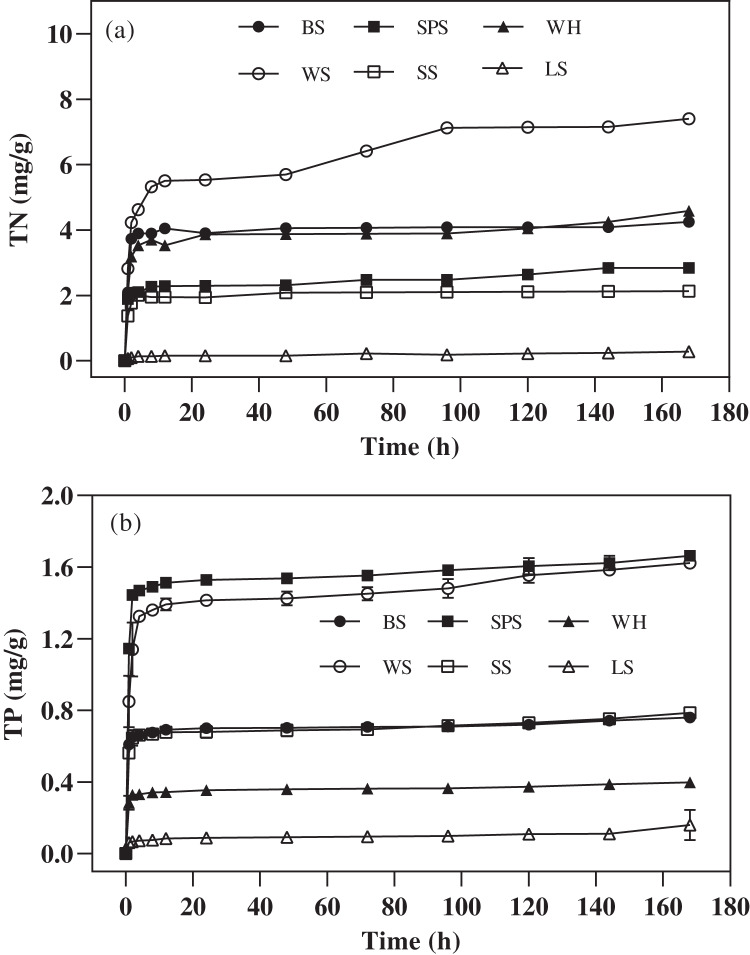
The TN and TP released by six agricultural wastes. Each data point represents the average content of TN (A) and TP (B) released by each agricultural waste during the 168-h reaction. BS, banana stalk; SS, sorghum stalk; SPS, sweet potato stalk; WH, wheat husk; LS, loofah sponge; WS, watermelon skins.

The amount and change trend of secondary pollutants release of each agricultural waste mixed with ultrapure water are shown in Fig. 2, The TN and TP were rapidly released from the six agricultural wastes in the first 2 h, after which the release rate dropped to very low levels (2–168 h). Figure 2A shows that the amount of TN released depended on the type of agricultural waste. The highest amount of TN was released from WS, followed by WH, BS, SPS, SS, and LS (7.41 ± 0.07, 4.58 ± 0.71, 4.25 ± 0.07, 2.84 ± 0.08, 2.13 ± 0.09, and 0.28 ± 0.01 mg/g, respectively). Under practical application, excessive TN release from agricultural waste may lead to incomplete denitrification and excessive nitrogen emissions. The largest accumulative concentration of released TP was achieved by WS and SPS leachate (1.62 ± 0.01 and 1.66 ± 0.01 mg/g, respectively) while the lowest occurred in LS leachate (0.16 ± 0.07 mg/g). As a result, LS has the lowest risk of secondary pollution. In addition, visual observation showed that the chroma in the leachate of WS and SPS was heavier than that of the others.

### Surface characteristics before and after carbon release

**Figure 3:**
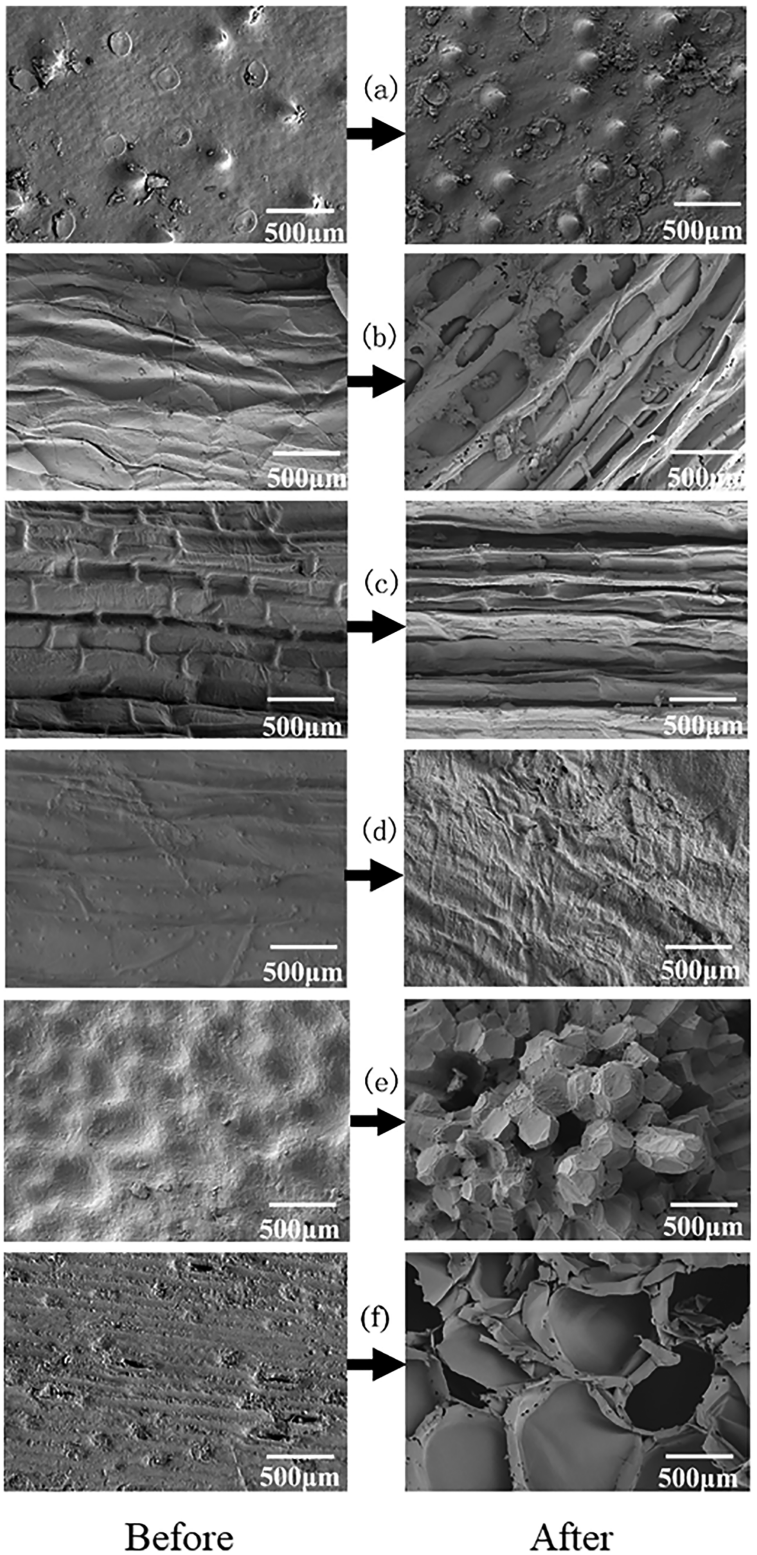
SEM images of six agricultural wastes before and after the carbon release experiment. Each figure indicates the surface structure before and after carbon release of each agricultural waste, with a magnification of 500 times. BS, banana stalk; SS, sorghum stalk; SPS, sweet potato stalk; WH, wheat husk; LS, loofah sponge; WS, watermelon skins. (A) WH; (B) SS; (C) SPS; (D) LS; (E) BS; (F) WS.

The surface morphologies of agricultural waste will affect the growth and reproduction of microorganisms. By analyzing the microstructure changes of agricultural waste before and after the immersion, it is helpful to evaluate its possibility as carbon source and microbial carrier. As shown in Fig. 3, the raw LS, BS and WS were comprised of smooth fibrils with low porosity, while the surfaces of fresh SS and SPS were uneven. The surface of WH was dense, with conical protrusions. The microscopic surface morphologies of the six agricultural wastes changed after soaking. WS lost its original structure, indicating that it had the most soluble substances, which is also one of the reasons for the highest COD release. For this reason, we conclude that WS is unsuitable for long-term carbon release. The surfaces of BS, SS, and SPS were uneven after soaking, with obvious void structures. This is conducive to the growth and adhesion of bacterial, but it is also the reason for the excessive release of COD. In contrast, WH maintained a dense surface structure, which limited the penetration of microorganisms into the WH. The hydrolysis of LS was relatively moderate, and the surface roughness increased. This was conducive to the attachment and growth of bacteria.

### FTIR analysis

**Figure 4:**
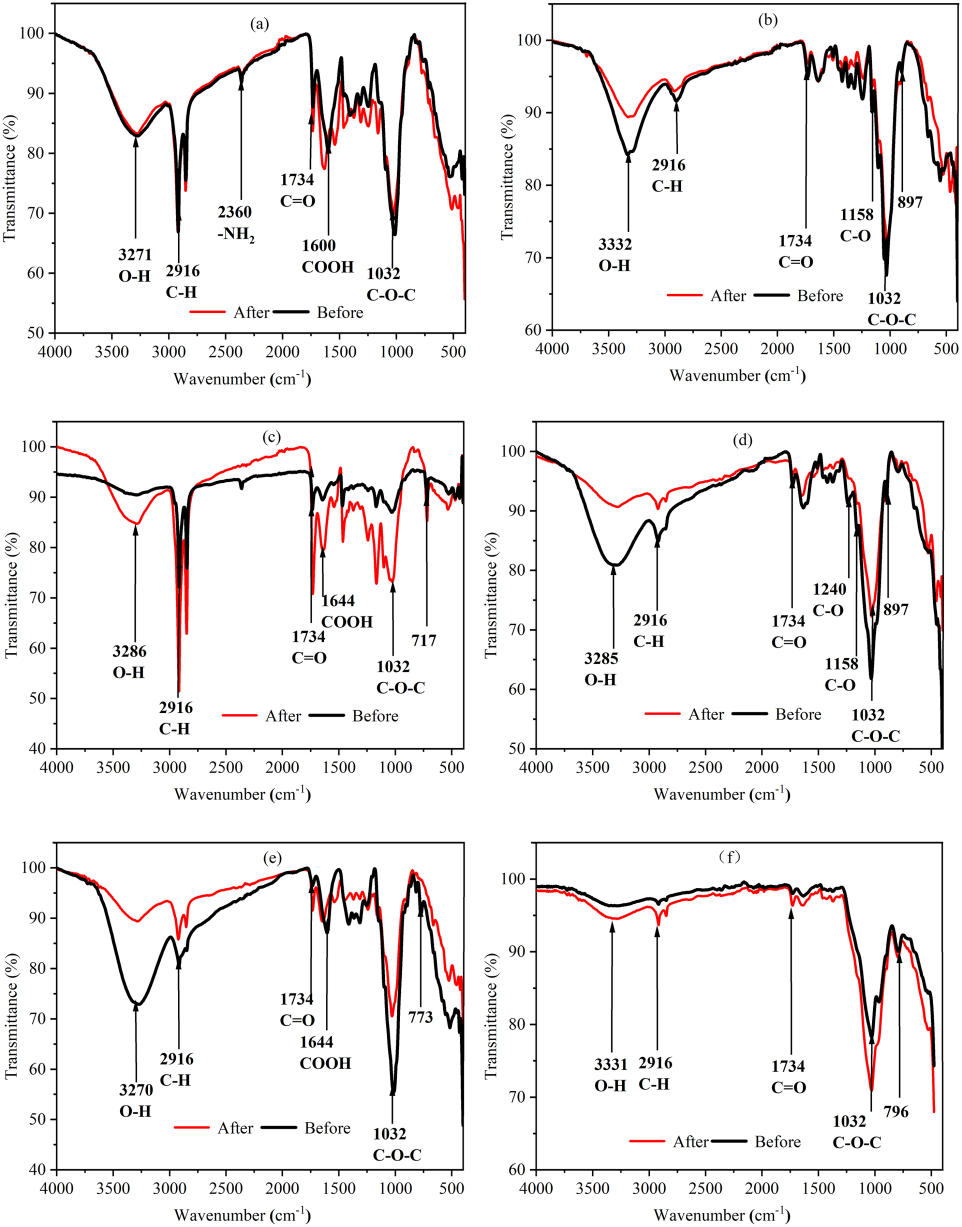
FTIR spectra of six agricultural wastes before and after carbon release. Each figure indicates the transmittance (%) of each agricultural waste before carbon release (black line) and after carbon release (red line) at different wavenumbers (cm^−1^). BS, banana stalk; SS, sorghum stalk; SPS, sweet potato stalk; WH, wheat husk; LS, loofah sponge; WS, watermelon skins. (A) SPS; (B) LS; (C) WS; (D) WH; (E) BS; (F) SS.

By analyzing the FTIR results of agricultural wastes before and after soaking, it is helpful to understand the changes of their composition and evaluate the structural stability of agricultural wastes. Figure 4 shows that the absorption peaks of each agricultural waste had the same wavenumber before and after the soaking assay. However, the absorbance of the functional groups differed before and after the soaking assay due to the different contents of lignin, cellulose, and hemicellulose in each agricultural waste. BS has higher absorbance to hydrophilic functional groups such as -OH and C-O-C, indicating that it is conducive to the growth of microorganisms. Further, the strong absorbance of BS to C-O-C also indicates that it contains more cellulose for microbial hydrolysis, while the cellulose content of WS is lower. In addition, the difference of FTIR spectra of WS before and after carbon release is the largest, which also shows that its structure is unstable and is not conducive to continuous carbon release, which is consistent with the SEM results. On the contrary, SPS and LS had the most similar FTIR spectrum before and after soaking, indicating that their structure remained the most stable. The higher absorption peaks of SPS and WS at 2,916 cm^−1^ were caused by the C-H stretching vibration of saturated hydrocarbons, indicating that they contain more organic compounds, which may lead to higher carbon release, which is consistent with the COD release results. BS, LS, and WH experienced a reduction in the absorption of infrared light below 900 cm^−1^ after soaking, indicating that the carbon release process led to the destruction of many ring structures. However, SS experienced an increase in the absorption of infrared light below 900 cm^−1^ and in the range of 3,000 to 3,500 cm^−1^, indicating the presence of a large amount of bound water on their surfaces. After soaking, the COOH bond absorption peak of WS increased and the lignin content increased, which was caused by the release of a large amount of water-soluble organic matter.

### Nitrogen removal

#### Variations in NO_3_^−^-N, NO_2_^−^-N, TN, TP, and COD concentrations

**Figure 5:**
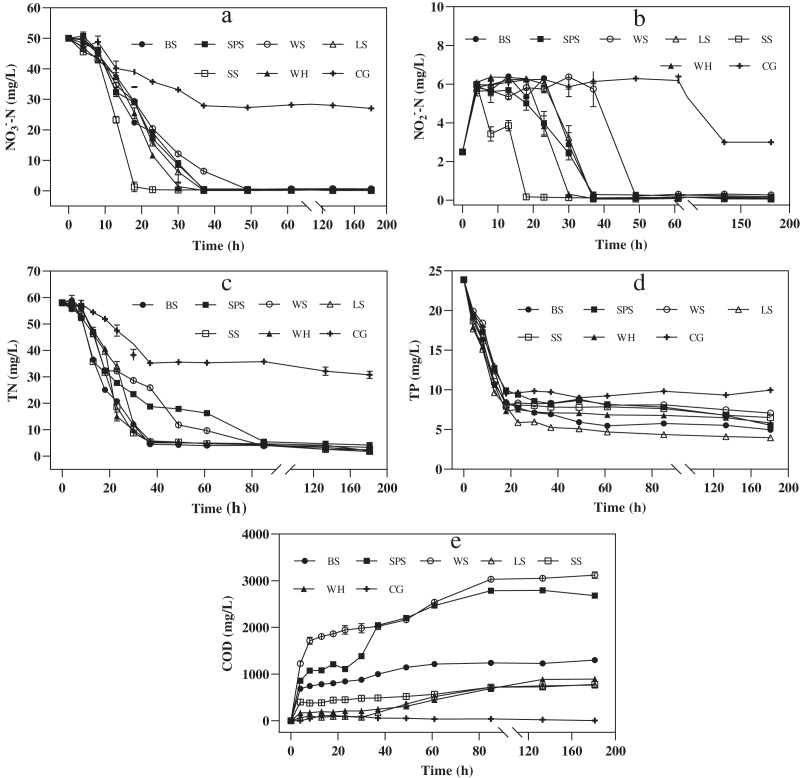
Denitrification performance of each agricultural waste during nitrogen removal experiment. The changes of NO_3_^−^-N concentration (A), NO_2_^−^-N concentration (B), TN concentration (C), TP concentration (D) and COD concentration (E) of six agricultural wastes. BS, banana stalk; SS, sorghum stalk; SPS, sweet potato stalk; WH, wheat husk; LS, loofah sponge; WS, watermelon skins; CG, Control group.

To investigate how the agricultural wastes could act as a carbon source to enhance denitrification, a denitrification experiment was carried out for 181 h. The COD in the denitrification process has similar characteristics to that of the carbon release process, as shown in Fig. 5E. WS had the highest cumulative COD, followed by SPS, BS, WH, SS, LS, and CG (3,119.33 ± 54.7, 2,679.67 ± 9.03, 1,299.67 ± 13.10, 892.17 ± 4.59, 780.17 ± 4.59, 763.33 ± 40.84, and 2.67 ± 1.89 mg/L, respectively). Compared to the soaking experiment, denitrification showed an increase in COD for all the agricultural wastes, except for BS. This shows that the addition of denitrifying sludge accelerated the release of carbon. In addition, compared with CG, during denitrification, all of the agricultural wastes significantly increased the nitrate and nitrite removal efficiency by 100% (Fig. 5A and Fig. 5B). This indicates that each agricultural waste provides sufficient organic matter to achieve complete denitrification. One-way analysis of variance showed that there was no significant difference in COD between SS and LS (*P* = 0.53), but there were significant differences among the other wastes (*P* = 0.0000–0.0009), indicating that SS and LS have the lowest risk of excessive accumulated COD.

Figure 5B shows that nitrate removal mainly occurred in the initial 37 h. NO_2_^−^-N accumulated in this stage as an intermediate product of denitrification. From 0 to 4 h, the NO_2_^−^-N concentrations increased rapidly and then, stabilized at approximately 6.00 mg/L. The NO_2_^−^-N concentrations decreased when the NO_3_^−^-N concentrations were low, until all the NO_2_^−^-N was removed. Nitrate was completely removed from SS after 23 h; LS, WH, BS and SPS after 37 h, and WS after 49 h, with corresponding nitrate removal rates of 2.16, 1.35, 1.35, 1.34, 1.34, and 1.01 mg NO_3_^−^-N/(L**·**h), respectively. Hence, the sequence of the complete removal of nitrate was the same as that of nitrite. For SS, the nitrate removal rate was higher than that of the other agricultural wastes, indicating that the denitrification start-up time was shorter. The shape of the TN removal curves was similar to that of the NO_3_^−^-N removal curves (Fig. 5C). After 181 h, the TN removal efficiencies of SS, WH, LS, BS, WS, and SPS were 96.94 ± 0.35, 96.52 ± 0.53, 96.23 ± 0.45, 95.72 ± 0.16, 94.20 ± 0.56, and 92.76 ± 1.10%, respectively. SS, LS, WH, and BS also had significantly higher TN removal efficiencies than SPS (*P* = 0.0006–0.0072). Furthermore, LS had the highest TP removal efficiency, followed by BS, SPS, WH, SS, and WS (83.35 ± 0.17%, 79.19 ± 0.21%, 76.81 ± 0.16%, 75.60 ± 0.18%, 72.73 ± 0.28% and 70.40 ± 0.23%, respectively). The differences in these values was determined to be significant (*P* = 0.0000) (Fig. 5D).

### Kinetics analysis of denitrification

The kinetics of the NO_3_^−^-N removal process (the stage of NO_3_^−^-N concentration above 0.5 mg/L) is described in a simple form by Eqs. (6) and (7) and is presented in Table 3. The results showed that there was a higher correlation between the denitrification process and zero-order kinetics (R^2^ > 0.9) than first-order kinetics (0.71 < R^2^ < 0.89). The *k*_*1*_ value of SS (−2.50) was significantly lower than that of other agricultural wastes, indicating that the start-up time of the denitrification of SS was shorter. Based on the above results, we conclude that SS is suitable for the fast start-up stage of denitrification, while LS is suitable for long-lasting denitrification.

**Table 3:** Kinetic equation fitting of denitrification process.

Carbon source	Zero-order equation	*k*_*1*_	R^2^	First-order equation	*k*_*2*_	R^2^
BS	*c*_t_ = −1.4449 *t* + 52.508	−1.44	0.98	ln (*c*_t_) = −0.1102 *t* + 4.63	−0.1102	0.74
SPS	*c*_t_ = −1.4739 *t* + 53.761	−1.47	0.98	ln (*c*_t_) = −0.0948 *t*+ 4.46	−0.0948	0.89
WH	*c*_t_ = −1.5931 *t* + 54.022	−1.59	0.96	ln (*c*_t_) = −0.1508 *t* + 4.92	−0.1508	0.83
WS	*c*_t_ = −1.1447 *t* + 50.792	−1.14	0.96	ln (*c*_t_) = −0.0858 *t* + 4.53	−0.0858	0.86
SS	*c*_t_ = −2.4967 *t* + 54.826	−2.50	0.93	ln (*c*_t_) = −0.2228 *t* + 4.79	−0.2228	0.84
LS	*c*_t_ = −1.4706 *t* + 53.039	−1.47	0.98	ln (*c*_t_) = −0.1375 *t* + 4.89	−0.1375	0.71

**Note:**

Each data used in the fitting equation is the average nitrate concentration of each agricultural waste in the stage before nitrate is completely removed (NO_3_^−^-N > 0.5 mg/L). Where *c_t_* is the nitrate concentration at *t*, mg/L; *t* is time, h; *k_1_* is the zero-order rate constant; *k_2_* is the first-order rate constant. BS, banana stalk; SS, sorghum stalk; SPS, sweet potato stalk; WH, wheat husk; LS, loofah sponge; WS, watermelon skins.

## Discussion

COD indicates the amount of oxidant that is consumed by reducing substances that are easily oxidized by strong oxidants and can be used to characterize the carbon release capacity of different agricultural wastes. The six agricultural wastes in this study, with the exception of LS, had two stages in the carbon release processes. These stages included the carbon release start-up stage (0–4 h) and the carbon release stable stage (4–168 h), with cumulative CODs of 79.68–535.68 mg/g. Ling et al. (2021) reported that the carbon release process of rice straw, wheat straw, corn stalk, corncob, soybean stalk, and soybean could also be divided into two phases; the quick release stage (0–6 h) and release stable stage (6–120 h), with cumulative CODs of 227.69–1,680.84 mg/(g**·**L). Similar results have also been reported for corn straw, reed, cattail, rice husk, loofah, cotton and coconut fiber (Yang & Wang, 2013; Lyu et al., 2020; Tao et al., 2020; Zheng et al., 2021). This two-stage release process can be attributed to the following reasons: in the first stage, a large amount of small and water-soluble molecular organic matter in agricultural wastes is dissolved with swelling; however, in the second stage, insoluble organic matter, such as cellulose and lignin, are slowly released into the solution under the action of bacteria hydrolysis (Cao et al., 2016). COD release in LS increased slowly and then, maintained a stable release rate throughout the carbon release process, attributing to the presence of less small and water-soluble molecular organics than the other agricultural wastes. LS also had lower *c*_m_ and the higher *t*_*1/2*_ values than the other agricultural wastes, indicating that the amount and velocity of carbon release was lower. In this study, WS had a higher *c*_m_ value than the other wastes, indicating that it contained more small and water-soluble molecular organics. This makes it a bad carbon source for the initial denitrification process, due to its excessive COD. SEM and FTIR results also demonstrate that the structure of WS was seriously damaged after carbon release, indicating that more water-soluble substances were dissolved as the cellulose degraded. This indirectly explains why WS had the highest COD release. It should be noted that the hydrolysis plays a key role in carbon release process, which is mainly caused by hydrolytic enzymes excreted by the microorganisms. In this study, we used agricultural wastes mixed with pure water in carbon source release experiment, which is a limitation for carbon release, so the application research of these agricultural wastes using real field aquaculture wastewater should be carried out in the next study.

In this study, the six agricultural wastes significantly enhanced the nitrate removal process compared to the control group. Denitrification rates ranged from 1.01 to 2.16 mg/(L**·**h), which were higher than the previously studied rates of rice straw (0.83 mg/(L**·**h)) (Zhu et al., 2021), reed (0.33 mg/(L**·**h)), cattail (0.29 mg/(L**·**h)) (Zheng et al., 2021), and hemp fiber (1.2 mg/(L**·**h)) (Yang & Wang, 2013). However, they were lower than the denitrification rate of corncob, which Zhong et al. (2019) found to reach 6.25 mg/(L**·**h). Previous studies have shown that the denitrification rate is not only affected by COD release but is also related to the following factors: (1) iron and manganese release from agricultural waste (Labbe, Parent & Villemur, 2003); (2) the relative proportion of COD from organics easily utilized by microorganisms (such as volatile fatty acids, carbohydrates, and sugars) (Gao et al., 2020); (3) the quality of the inoculated sludge, its reaction conditions, and the characteristics of the wastewater (Xiong et al., 2019). For example, Yang et al. (2015) found that although the COD release of rice straw is lower than that of loofah and corncob, it had higher denitrification rates. In this study, SEM images showed that the dense cavity structure on the surface of SS after immersion can provide a larger breeding space for microorganisms, which promotes the decomposition and utilization of the carbon source; this allows nitrate to be removed first. The relatively dense surface structures of WH and LS delayed the occurrence of this process. SEM and FTIR results demonstrate that the structure of WS was the most severely damaged after immersion, resulting in the loss of attachment and growth conditions of microorganisms. In addition, its high TN release also increased the nitrate load, resulting in the longest nitrate removal time.

Nitrite is the intermediate product of denitrification, and its concentration increased with a decrease in nitrate content. NO_3_^−^-N reductase competes with NO_2_^−^-N reductase for matrix electrons and inhibits the activity of NO_2_^−^-N reductase. Therefore, nitrite concentration will continue to be reduced when nitrate concentration low (Gan, Zhao & Ye, 2019). As a result, the nitrite removal sequence of the six agricultural wastes was the same as that of nitrate, which is similar to the results of Gao et al. (2020). After 181 h, nitrate and nitrite were completely removed, indicating that the six agricultural wastes released sufficient organic matter to achieve complete denitrification. However, the TN removal efficiency of WS and SPS were relatively low. On the one hand, the excessive COD release of WS and SPS led to a higher number of electron donors in the water than the receptors (nitrate). Furthermore, the NO_3_^−^-N was dissimilated and reduced to NH_4_^+^-N, resulting in a lower TN removal efficiency. Similar results were found in a study by Ling et al. (2021). On the other hand, the higher TN release of WS led to a lower TN removal efficiency, which has also been observed in the study by Guan et al. (2019). In addition, the different carbon, nitrogen, and phosphorus release of the six agricultural wastes led to different C/N/P ratios in the water. Huang et al. (2019) and Zhen et al. (2019) confirmed that different C/N/P ratios lead to different TN and TP removal efficiencies. In future research, we can further explore the effect of the C/N/P ratios on the denitrification performance of six agricultural wastes.

In this study, the six types of agricultural wastes were found to have excellent denitrification performance and completely removed both nitrate and nitrite. Many previous studies have showed similar improved denitrification performance by adding agricultural waste to a denitrification system. When woodchips were added to a baffle subsurface flow constructed wetland, the removal rate of nitrate reached 63.6–96.1% (Yuan et al., 2020). Lu et al. (2021) reported that they designed a composite filter bed reactor integrating sulfur, iron (II) and shaddock peel, which can improve the denitrification efficiency up to more than 90%. Li, Sun & Song (2019) has shown that maize cobs enhance nitrogen removal from effluents of marine recirculating aquaculture system in saline constructed wetlands. Loofa sponge had the lowest pollutant release rate and cumulative COD, as well as the highest removal rates of TP and TN, thus it is the most suitable waste to use as a solid carbon source for aquaculture wastewater treatment with a low C/N ratio. However, in the future, the specific impacts of each of the six agricultural wastes as external carbon sources on the aquaculture wastewater treatment system in practical application needs to be further studied.

## Conclusions

(1) The COD release of WH, SS, SPS, WS, BS and LS ranged from 37.74 to 535.68 mg/g, with WS having the highest carbon release. Of these agricultural wastes, LS was found to have lower risks of excessive carbon release. Of the leachate from the six agricultural wastes, WS and SPS had the highest TN and TP content, respectively. Visual observation showed that the chroma in the leachate of WS and SPS was heavier than that of the others. In contrast, LS had the lowest TN and TP released amount, as well as the lowest risk of secondary pollution.
(2) The carbon release process of the six agricultural wastes conforms to the R-P and second-order kinetic equations, with the main carbon release mechanism being diffusion. LS had the minimum *c*_*m*_ and the maximum *t*_*1/2*_ values, which is conducive to continuous and stable carbon release, and avoids exceeding the effluent COD. SEM and FTIR results showed that the structure of WS was seriously damaged after immersion in water and did not have the ability to continuously release carbon. However, the degree of hydrolysis of LS was relatively moderate, and its roughness increased after carbon release, which is conducive to microbial adhesion.
(3) In the 181-h denitrification process, nitrate and nitrite can be completely removed from the six agricultural wastes. The process also conforms to the zero-order and first-order kinetic equations. SS, LS, and WH had the highest TN removal efficiencies, while LS had the highest TP removal efficiency and the lowest accumulated COD.

As a result, LS was more suitable as a solid carbon source for denitrification than the other five agriculture wastes. In practical applications, long-lasting carbon release capacity, high TN and TP removal efficiency, and low excessive carbon release and secondary pollution risks can be obtained in the aquaculture wastewater treatment if LS is applied as the external carbon source.

## Supplemental Information

10.7717/peerj.13339/supp-1 Supplemental Information 1 Raw data of COD released, FTIR spectra, NO_3_^−^-N, NO_2_^−^-N, TN, TP and COD concent of six agricultural wastes. Raw data for Figs. 1 and 4 and Tables 2 and 3; FTIR spectra.

10.7717/peerj.13339/supp-2 Supplemental Information 2 Raw data for SEM images of six agricultural wastes before and after the carbon release experiment. Each figure indicates the surface structure before (left) and after (right) carbon release of each agricultural waste, with a magnification of 500 times. BS, banana stalk; SS, sorghum stalk; SPS, sweet potato stalk; WH, wheat husk; LS, loofah sponge; WS, watermelon skins.

## Additional Information and Declarations

### Data Availability

The following information was supplied regarding data availability: The raw measurements are available in the Supplemental Files.

## References

[ref-1] Cao X, Li Y, Jiang X, Zhou P, Zhang J, Zheng Z (2016). Treatment of artificial secondary effluent for effective nitrogen removal using a combination of corncob carbon source and bamboo charcoal filter. International Biodeterioration & Biodegradation.

[ref-2] Contrera RC, Silva KC, Morita DM, Rodrigues JAD, Zaiat M, Schalch V (2014). First-order kinetics of landfill leachate treatment in a pilot-scale anaerobic sequence batch biofilm reactor. Journal of Environmental Management.

[ref-4] Fang JH, Jiang ZJ, Jansen HM, Hu FW, Fang JG, Liu Y, Gao YP, Du M (2017). Applicability of *Perinereis aibuhitensis* Grube for fish waste removal from fish cages in Sanggou Bay, P. R. China. Journal of Ocean University of China.

[ref-3] Food and Agriculture Organization of the United Nations (FAO) (2020). The state of world fisheries and aquaculture 2020.

[ref-5] Fu DF, Kai H, Singh RP, Ducoste JJ (2014). Enhanced nitrogen removal by rice husk amended dynamic membrane bioreactors. Journal of Environmental Engineering.

[ref-6] Gan Y, Zhao Q, Ye Z (2019). Denitrification performance and microbial diversity of immobilized bacterial consortium treating nitrate micro-polluted water. Bioresource Technology.

[ref-7] Gao YD, Guo L, Shao MY, Hu FW, Wang GC, Zhao YG, Gao MC, Jin CJ, She ZL (2020). Heterotrophic denitrification strategy for marine recirculating aquaculture wastewater treatment using mariculture solid wastes fermentation liquid as carbon source: optimization of COD/NO_3_^−^-N ratio and hydraulic retention time. Bioresource Ttechnology.

[ref-8] Guan X, Ji G, Xu S, Yun Y, Liu H (2019). Selection of agricultural straws as sustained-release carbon source for denitrification in a drawer-type biological filter. Water, Air, & Soil Pollution.

[ref-9] Huang L, Guo HY, Liu XF, Zhang SN (2019). Effects of influent C/N/P on denitrifying phosphorus removal efficiency and carbon source conversion and utilization. Environmental Pollution & Control.

[ref-10] Huang XF, Liu X, Shang JJ, Feng Y, Liu J, Lu LJ (2012). Pretreatment methods for aquatic plant biomass as carbon sources for potential use in treating eutrophic water in subsurface-flow constructed wetlands. Water Science and Technology.

[ref-11] Huang YY (2017). Research progress of wastewater treatment by agricultural wastes as biological adsorbent. Applied Chemical Industry.

[ref-12] Jing L, Sun Y, Wang H, Liu W, Wang D (2019). Denitrification in simulated groundwater using lignite as a solid-phase organic carbon source. TECNOLOGIA Y CIENCIAS DEL AGUA.

[ref-13] John EM, Krishnapriya K, Sankar TV (2020). Treatment of ammonia and nitrite in aquaculture wastewater by an assembled bacterial consortium. Aquaculture.

[ref-14] Khursheed A, Gaur RZ, Sharma MK, Tyagi VK, Khan AA, Kazmi AA (2018). Dependence of enhanced biological nitrogen removal on carbon to nitrogen and rbCOD to sbCOD ratios during sewage treatment in sequencing batch reactor. Journal of Cleaner Production.

[ref-15] Labbe N, Parent S, Villemur R (2003). Addition of trace metals increases denitrification rate in closed marine systems. Water Research.

[ref-16] Li C, Li J, Liu G, Deng Y, Zhu S, Ye Z, Shao Y, Liu D (2019). Performance and microbial community analysis of Combined Denitrification and Biofloc Technology (CDBFT) system treating nitrogen-rich aquaculture wastewater. Bioresource Technology.

[ref-17] Ling Y, Yan GK, Wang HY, Dong WY, Wang H, Chang Y, Chang M, Li CY (2021). Release mechanism, secondary pollutants and denitrification performance comparison of six kinds of agricultural wastes as solid carbon sources for nitrate removal. International Journal of Environmental Research and Public Health.

[ref-18] Li M, Sun LL, Song XF (2019). Adding maize cobs to vertical subsurface flow constructed wetlands treating marine recirculating aquaculture system effluents: carbon releasing kinetics and intensified nitrogen removal. Bioresource Technology.

[ref-19] Liu M, Li B, Xue Y, Wang H, Yang K (2017a). Constructed wetland using corncob charcoal substrate: pollutants removal and intensification. Water Science and Technology.

[ref-20] Liu Y, Liu C, Nelson WC, Shi L, Xu F, Liu Y, Yan A, Zhong L, Thompson C, Fredrickson JK, Zachara JM (2017b). Effect of water chemistry and hydrodynamics on nitrogen transformation activity and microbial community functional potential in hyporheic zone sediment columns. Environmental Science & Technology.

[ref-21] Lu XJ, Wan YL, Zhong ZX, Liu B, Zan FX, Zhang FG, Wu XH (2021). Integrating sulfur, iron (II), and fixed organic carbon for mixotrophic denitrification in a composite filter bed reactor for decentralized wastewater treatment: performance and microbial community. Science of the Total Environment.

[ref-22] Lyu CJ, Song YH, Gao HJ, Yao CY, Lu JX, Li XJ (2020). Promoting effects of corn straw and exceed sludge as carbon sources on denitrification of constructed wetlands.

[ref-23] Ministry of Ecology & Environment of the People’s Republic of China, National Bureau of Statistics of China, Ministry of Agriculture and Rural Affairs of the People’s Republic of China (2020). The Second National Pollution Source Census Bulletin.

[ref-24] Ryu HD, Lee SI (2009). Comparison of 4-Stage Biological Aerated Filter (BAF) with MLE process in nitrogen removal from low carbon-to-nitrogen wastewater. Environmental Engineering Science.

[ref-25] Tao ZK, Jing ZQ, Wang Y, Tao MN, Luo H (2020). Higher nitrogen removal achieved in constructed wetland with polyethylene fillers and NaOH-heating pre-treated corn stalks for advanced treatment of low C/N sewage. Environmental Science and Pollution Research.

[ref-26] Tu X, Xiao B, Xiong J, Chen X (2010). A simple miniaturised photometrical method for rapid determination of nitrate and nitrite in freshwater. Talanta.

[ref-27] Wen Y, Chen Y, Zheng N, Yang DH, Zhou Q (2010). Effects of plant biomass on nitrate removal and transformation of carbon sources in subsurface-flow constructed wetlands. Bioresource Technology.

[ref-28] Xiong R, Yu X, Yu L, Peng Z, Cheng L, Li T, Fan P (2019). Biological denitrification using polycaprolactone-peanut shell as slow-release carbon source treating drainage of municipal WWTP. Chemosphere.

[ref-29] Xiong R, Yu X, Zhang Y, Peng Z, Yu L, Cheng L, Li T (2020). Comparison of agricultural wastes and synthetic macromolecules as solid carbon source in treating low carbon nitrogen wastewater. Science of the Total Environment.

[ref-30] Xu ZS, Dai XH, Chai XL (2019). Effect of temperature on tertiary nitrogen removal from municipal wastewater in a PHBV/PLA-supported denitrification system. Environmental Science and Pollution Research.

[ref-31] Yang F, Wang HL (2013). Slow-release carbon source composite materials’ preparation and used for denitrification in groundwater. Advanced Materials Research.

[ref-32] Yang XL, Jiang Q, Song HL, Gu TT, Xia MQ (2015). Selection and application of agricultural wastes as solid carbon sources and biofilm carriers in MBR. Journal of Hazardous Materials.

[ref-33] Yuan C, Zhao F, Zhao X, Zhao Y (2020). Woodchips as sustained-release carbon source to enhance the nitrogen transformation of low C/N wastewater in a baffle subsurface flow constructed wetland. Chemical Engineering Journal.

[ref-34] Zhang K, Yu DG, Li ZF, Xie J, Wang GJ, Gong WB, Yu EM, Tian JJ (2020). Influence of eco-substrate addition on organic carbon, nitrogen and phosphorus budgets of intensive aquaculture ponds of the Pearl River, China. Aquaculture.

[ref-35] Zhen JY, Yu DS, Wang XX, Chen GH, Du YQ, Yuan MF, Du SM (2019). Effect of the influent C/P ratio on the nutrient removal characteristics of the SNEDPR system. Environmental Science.

[ref-36] Zheng Y, Cao T, Zhang Y, Xiong J, Dzakpasu M, Yang D, Yang Q, Liu Y, Li Q, Liu S, Wang X (2021). Characterization of dissolved organic matter and carbon release from wetland plants for enhanced nitrogen removal in constructed wetlands for low C-N wastewater treatment. Chemosphere.

[ref-37] Zheng XW, Zhang SY, Zhang JB, Huang DY, Zheng Z (2018). Advanced nitrogen removal from municipal wastewater treatment plant secondary effluent using a deep bed denitrification filter. Water Science and Technology.

[ref-38] Zhou BB, Duan JJ, Xue LH, Zhang JW, Yang LZ (2019). Effect of plant-based carbon source supplements on denitrification of synthetic wastewater: focus on the microbiology. Environmental Science and Pollution Research.

[ref-39] Zhong H, Cheng Y, Zhang HW, Shao YL, Zeng GM (2019). Performance of Corncob-based solid phase denitrification system: a column study. Journal of Hunan University (Natural Sciences).

[ref-40] Zhu HX, Zhang SN, Peng YX, Xiao J, Liu F, Xiao RL, Dai GJ, Zhu XJ (2021). Study on carbon release characteristics and denitrification performance of different solid carbon sources. Research of Agricultural Modernization.

[ref-41] Zhu SM, Deng YL, Ruan YJ, Guo XS, Shi MM, Shen JZ (2015). Biological denitrification using poly (butylene succinate) as carbon source and biofilm carrier for recirculating aquaculture system effluent treatment. Bioresource Technology.

